# Effect of homogenizer pressure and temperature on physicochemical, oxidative stability, viscosity, droplet size, and sensory properties of Sesame vegetable cream

**DOI:** 10.1002/fsn3.680

**Published:** 2019-02-07

**Authors:** Sara Amiri Samani, Mohammad Hadi Naji

**Affiliations:** ^1^ Department of Food Science and Technology Shahrekord Branch Islamic Azad University Shahrekord Iran; ^2^ Department of Food Science and Technology Zarin Dasht Branch Islamic Azad University Fars Iran

**Keywords:** droplet size, homogenizer pressure, oxidative stability, vegetable cream, viscosity

## Abstract

In this study, the effects of homogenization pressure (125, 145, and 165 bars) and temperature (45, 60, and 75°C) on the properties of Sesame vegetable cream are investigated. The physical stability of cream was characterized by droplet size and syneresis, and chemical stability of it was evaluated by determining peroxide value and p‐anisidine. The results showed that the cream in the presence of high pressure and temperature treatment exhibits lower stability. At 75°C temperature and 165 bar, the vegetable cream had highest peroxide value (3.61) and p‐anisidine (2.16). However, pressure could protect the droplets against aggregation in the high pressure (165 bar) and greatly increased the physical stability. During increase in process parameters, the syneresis of cream was decreased with a rise of pressure and extension of temperature. The process condition in 145 bar and 60°C led to the high acceptability of vegetable cream.

## 1 INTRODUCTION

Emulsions are droplets suspensions of immiscible fluid dispersed in another fluid, such as water in oil (w/o‐emulsions) or oil in water (o/w emulsion) type. Emulsifiers, usually surface‐active components, help to form kinetically stable emulsions by reducing the water–oil interfacial tension or by forming a protective layer around the oil droplets (Abdolmaleki, Mohammadifar, Mohammadi, Fadavi, & Meybodi, 2016;  Hoffmann & Reger, 2014). Hydrocolloids act as emulsifying agents usually owning to their amphiphilic properties (Chuah, Kuroiwa, Kobayashi, & Nakajima, 2014).

Emulsions are usually classified according to the spatial distribution of the water and oil phases relative to each other. An oil in water emulsions is a system where the droplets of oil are dispersed within a water phase. The dominant functions of emulsions are their ability to contain lipophilic ingredients into food system. Thus, it is crucial to develop an emulsion that is most stable (Gahruie, Ziaee, Eskandari, & Hosseini, 2017). In the food industry, a homogenization process, which often subjects the liquids to mechanical agitation (e.g., high‐pressure valve homogenizers, high‐speed blenders, and colloid mills), is carried out to increase the stability of o/w emulsions (Perrier‐Cornet, Marie, & Gervais, 2005).

Pharmaceutical, chemical, biotechnology facilities and specially foods all use the high‐pressure homogenizer to emulsification, mix, disperse, and process their products. One of the new homogenizer improvements design homogenizer with high pressure. The pressure‐level increase permits to decrease the droplet size of the prepared emulsions and then to increase shelf life of the emulsion by reducing creaming rate (Fernandez‐Avila & Trujillo, 2017). In addition to the decrease in the average droplet diameter of products, ultra high‐pressure homogenization can also deflocculate clusters of primary fat droplet, and dispersing agglomerates uniformly. Low‐pressure homogenization cannot be able to increase the energy threshold necessary to break these clusters droplet. High‐pressure homogenization increases the activity of surface of the emulsifying component, which may grow the efficiency of the emulsion (ability of coating or penetration action) (Floury, Desrumaux, & Lardieres, 2000).

Moreover, a considerable change in the oil droplets dispersion state would be the emulsification of some immiscible liquids (oil and water), which also results in a considerable state of the water–oil interface (McClements, 2015; Walstra, 1983), and mainly, two types of ingredient can be adsorbed: amphiphilic component (such as proteins) and emulsifiers with low molecular weight (monoglycerides, lecithins, spans, tweens, etc.) (Burgaud, Dickinson, & Nelson, 1990; Ozturk & McClements, 2016). Emulsifiers with low molecular weight and proteins help the production and the emulsions stabilization. Proteins play two main roles: First, they lower surface tension between the interfaces that are applied during the process of emulsification, and second, they form a component layer surrounding the dispersed ingredient, which structurally stabilizes the emulsions by reducing the rate of coalescence (Walstra, 1983). In food emulsions, stability is usually received by the proteins application as the main stabilizer.

In the homogenizer, some processing conditions including shear stress, high pressure, and temperature can lead to a protein‐stabilizing properties deterioration (Ozturk, Argin, Ozilgen, & McClements, 2015). However, when it is already established that structure of protein, it is susceptible to be modified by high‐pressure method, and it is not known to what extent the new technology, such as homogenization with dynamic high pressure, allows systematic modification of the texture and rheology of protein‐stabilized emulsions. Moreover, only the high hydrostatic pressure effects on the structure of the protein in aqueous solution have received considerable attention over the recent few years (Dickinson & Pawlowsky, 1996; Zhou et al., 2016). Consequences on globular proteins are that disrupts of high pressure the quaternary structure and tertiary. It is also known that globular protein unfolding is partly due to secondary structure changes (β‐sheets, α‐helices). As with thermal treatment, the globular protein partial denaturation used of hydrostatic high pressure leads to aggregation (Dumay, Kalichevsky, & Cheftel, 1994; He, Mao, Gao, & Yuan, 2016). Dumay, Lambert, Funtenberger, and Cheftel (1996) have reported emulsion pressure‐induced rheological modifications due to aggregation of β‐lactoglobulin. Galazka, Dickinson, and Ledward (1996) showed that high‐pressure process (up to 800 MPa) of the globular protein β‐lactoglobulin leads to a reduction in the capacity of emulsifying and a decrease in the fine emulsion stability made at pH 7 with 20 vol.% oil and 0.4 w.w.b% protein. The author reported this result as a low of emulsifying efficiency due to pressure‐induced unfolding followed by aggregation. Qualitatively similar effects of high‐pressure process on emulsifying characterization were also showed when β‐lactoglobulin was replaced with commercial WPC (Galazka, Ledward, Dickinson, & Langley, 1995).

The aim of this study was to evaluate the effect of homogenizer pressure (125, 145, and 165 bar) and temperature (45, 60, and 75°C) on physicochemical, oxidative stability, viscosity, droplet size, and sensory properties of vegetable cream.

## 2 MATERIALS AND METHODS

### 2.1 Materials and chemicals

Milk powder with 30% w/w protein, 0%‐0.2% fat, and 96% dry matter was prepared from the Fars Pegah Dairy Company (Shiraz, Iran). Sesame oil was purchased from Narges Oil Company (Shiraz, Iran). Monodiglyceride and BK were prepared from Danisco (China) Co., Ltd. (Kunshan, China).

### 2.2 Emulsion preparation

Oil in water emulsion contains 30% (w/w) fat, which was produced using monodiglycerides emulsifier (1%), BK stabilizer (0.04%), and milk powder (3) to 9 runs by factorial model. Milk powder and BK were mixed in distilled water. Monodiglycerides were entirely liquated and mixed with premix of sesame oil. Emulsification was performed using a homogenizer (FBF Italia, Italy) in 125, 145, and 165 bar and temperature 45, 60, and 75°C. Before evaluation tests, the mixes were stored at 4°C for 24 h. Each formulation (100 g) was produced in three repetitions (Vanderghem, Danthine, Blecker, & Deroanne, 2007).

### 2.3 Titratable Acidity and pH measurement

pH and total titratable acidity were determined by Bemer, Limbaugh, Cramer, Harper, and Maleky (2016) standard approach, and the acidity was expressed according to the lactic acid percentage.

### 2.4 Serum loss measurement

Cream (10 ml) centrifuged at speed of 140 g for 5 min using a centrifuge (Phillips HR1565, China). Amount of Serum loss was reported according to the serum percentage.

### 2.5 Peroxide value (PV) and P‐Anisidine value measurement

Extraction of Lipid was performed and utilized to determine lipid oxidation and p‐anisidine values as chemical indicator. Both p‐anisidine value (AV) and peroxide value (PV) were determined according to the method described by Keramat, Golmakani, Aminlari, and Shekarforoush (2016). For PV evaluation, 5 g oil was added to 30 ml solvent (acetic acid and chloroform mixture), and then, approximately 5 ml potassium iodide was added. The mixture was mixed for 1 min. After that, 30 ml of double distilled water was mixed with mixture, and then, starch solution was also added. Titration was carried out using 0.01 *N* sodium thiosulfate solution to appearance a transparent color. In AV, 0.2 g of oil was mixed with 10 ml of trimethylpentane. The A_1_ absorbance was determined at 350 nm. One ml of 2.5 g/L p‐anisidine in acetic acid was mixed with 5 ml of the oil‐trimethylpentane dispersion. After 10 min, the A_2_ absorbance was determined. AV was optioned from these absorbance, and the oil mass (m) previously calculated: “10 × [1.2 × (A_2_ − A_1_)]/m”.

### 2.6 Color measurement

Colorimetry operation was conducted by the digital colorimetry method. Digital pictures were taken from the surface of samples using a digital camera (Canon, Model IXUS 230 HS, 14.0 Megapixels, Tokyo, Japan) under the same optical conditions. Resolution, contrast, and lightness of all images were set to 300 dots per inch (dpi), 62 (%), and 62 (%), respectively. All pictures were saved as JPEG format, and the differences in their colors were assessed using Adobe Photoshop CS 5 Software (Adobe Systems Inc., Beijing, China). Color indexes such as L* (lightness), a* (redness–greenness), and b*(yellowness–blueness) were determined and analyzed for each individual sample (Afshari‐Jouybari & Farahnaky, 2011).

### 2.7 Droplet size distribution

Droplet size and span were measured based on the method described by Nejadmansouri, Hosseini, Niakosari, Yousefi, and Golmakani (2016) with some modification. Static light scattering technique (at 20°C temperature) was used to evaluate the size of the droplet (Laser Diffraction Particle Size, SALD‐2101, Shimadzu, Japan). The average droplet size (*z*‐average) and span were reported. The width of the droplet size distribution was shown as a span of distribution: span = (d90 − d10)/d50, where d × 0 is the diameter corresponding to ×0 intensity on a relative cumulative droplet size distribution curve.

### 2.8 Sensory Evaluation

At least 20 members of a trained panelist group were selected from the department of food science and technology (Zarin Dasht Branch, Islamic Azad University, Fars, Iran) and performed sensory properties. This test was carried out based on 5‐point Hedonic scale method (1 = dislike extremely, 2 = dislike moderately, 3 = neither like nor dislike, 4 = like moderately, and 5 = like extremely). The creams for organoleptic properties were coded and given to panelists in order to evaluate the acceptance rated of taste, aroma, texture, color, general acceptance. For this aim, the samples were kept in 4°C for 1 week (Bemer et al., 2016).

### 2.9 Statistical analysis

All experiments were evaluated in the form of completely random blocks and repeated three times. Duncan test was used to compare means when the effect was significant. *p* < 0.05 was used as a level of significance. The data were shown in the form of mean and standard deviation. SAS software version 9.1 was applied for statistical analysis (SAS Institute Inc., 2000; Cary, NC, USA).

## 3 RESULTS AND DISCUSSIONS

### 3.1 Physicochemical properties

The effects of pressure and temperature on physicochemical change for the different sample were evaluated. As shown in Table 1, the acidity in all samples increased significantly by increasing the pressure, but temperature does not have any significant effect on acidity. To increase homogenization level, O_2_ amount in cream and oxidation rate and produce of fatty acids increased significantly. Marco‐Molés, Hernando, Llorca, and Pérez‐Munuera (2012) evaluated the effects of homogenization on dairy emulsion; they reported by increasing homogenization from 0 to 250 MPa, acidity increased slowly from 0.15 to 0.18. These parameters represent the hydrogen ion concentration and the acidity level of food.

**Table 1:** Effects of homogenizer pressure and temperature on physicochemical, oxidative stability of cream

	Homogenizer pressure	Temperature	Acidity	pH	Syneresis	Peroxide	p‐Anisidine
1	125	45	0.11 ± 0.01de	6.31 ± 0.02a	4.58 ± 0.23a	1.75 ± 0.19c	1.96 ± o.13b
2	125	60	0.11 ± 0.01dec	6.31 ± 0.04a	4.33 ± 0.18a	1.46 ± 0.43c	1.95 ± 0.05b
3	125	75	0.10 ± 0.01e	6.31 ± 0.06a	4.53 ± 0.27a	1.49 ± 0.20c	2.01 ± 0.12ab
4	145	45	0.12 ± 0.01bcde	6.23 ± 0.02a	2.24 ± 0.13c	2.51 ± 0.30b	2.05 ± 0.15ab
5	145	60	0.12 ± 0.01bcde	6.26 ± 0.03a	2.36 ± 0.20c	2.46 ± 0.26b	1.99 ± 0.04ab
6	145	75	0.12 ± 0.01abcd	6.24 ± 0.02a	2.46 ± 0.14bc	2.27 ± 0.25b	1.95 ± 0.10b
7	165	45	0.12 ± 0.01abc	6.50 ± 0.22a	2.76 ± 0.09b	3.45 ± 0.25a	1.93 ± 0.03b
8	165	60	0.13 ± 0.01ab	6.14 ± 0.04a	2.44 ± 0.07bc	3.35 ± 0.23a	1.90 ± 0.13b
9	165	75	0.13 ± 0.01a	6.16 ± 0.03a	2.58 ± 0.25bc	3.61 ± 0.23a	2.16 ± 0.07a

Values within each column with different letters are significantly different (*p *<* *0.05).

The abbreviations are the same as in table.

All values are mean ± standard deviation.

The result of pH showed pressure and temperature do not have any significant effect on pH. Hayes and Kelly (2003) evaluated the effects of homogenization on whole milk properties. Their findings showed that by an increase in pressure from 0 to 200 MPa, pH decreases significantly. They reported a decrease in pH due to reduction in droplet size and increase in lipolysis of fat.

Greater volumes (10 ml) of the cream (at 5°C) were produced and used within stability tests. For most practical utilization, it is necessary that emulsion‐based systems remain physically resistant of the product. The syneresis in all samples decreased significantly when the pressure increased and 145 bar is the best pressure for production of cream. By increasing the pressure ability of cream, production significantly increased and syneresis decreased. But temperature does not have any significant effect on syneresis. Chandrapala et al. (2016) evaluated the effect of homogenization on cream properties. Results showed that with an increase in homogenization, serum loss value significantly reduces to 40%. This phenomenon is due to increased amount of kappa casein on the surface droplet.

### 3.2 Oxidative stability

The delivery systems that are based on emulsion and utilized to encapsulate polyunsaturated oils or other lipophilic nutraceuticals should be able to protect them from oxidation reactions. O/W emulsion systems usually have an aqueous phase which contains prooxidants and antioxidants. This phase strongly affects the interactions between oil and water components. Additionally, the lipid oxidation reactions in such a system are complex and completely different from bulk lipid systems (Waraho, Mcclements, & Decker, 2011). In O/W emulsion systems, prooxidants are the most important transition metals (Mancuso, Mcclements, & Decker, 2000). Different amounts of temperature did not show significant effects on peroxide value. But, by increasing pressure, peroxide value significantly increases. Increase in homogenization increases the amount of oxygen in cream and reduces oxidative stability. Temperature and pressure do not have any significant effect on p‐anisidine of samples. Marco‐Molés et al. (2012) evaluated the effect of homogenization on emulsion properties based on dairy product. The results of this research showed that by increasing pressure from 0 to 250 MPa, k232 as an oxidative stability index has a slight increase from 2.70 to 2.91.

### 3.3 Color properties

**Figure 1:**
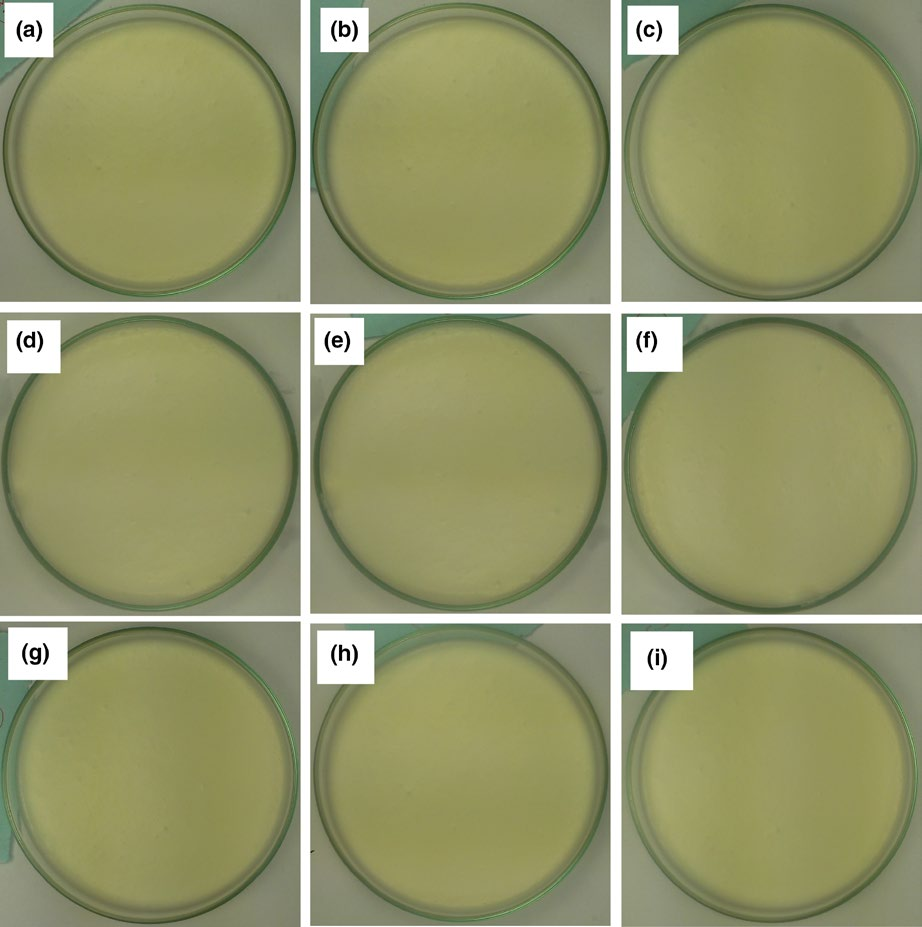
Vegetable creams produced at 125 bar and 45°C (a), 125 bar and 60°C (b), 125 bar and 75°C (c), 145 bar and 45°C (d), 145 bar and 60°C (e), 145 bar and 75°C (f), 165 bar and 45°C (g), 165 bar and 60°C (h), 165 bar and 75°C (i)

The effects of different concentration of homogenizer pressure and temperature on the color changes (L* (lightness), a* (redness–greenness) and b* (yellowness–blueness)) of vegetable cream treatments are shown in Table 2 and Fig 1. Comparison of L* values of treated samples showed a significant difference (*p* < 0.05). According to the results, the L* value in all samples increased significantly with increasing homogenizer pressure, but the L* value in all samples decreased significantly with increasing temperature. This change is due to the effect of pressure on droplet size distribution and viscosity. By increasing temperature and pressure, *L* value increases significantly (Quek, Chok, & Swedlund, 2007). Hayes and Kelly (2003) evaluated the effect of homogenizer pressure on properties of cow milk. They reported that with an increase in homogenizer pressure from 0 to 200 bar *L* value increases significantly, due to a decrease in droplet size distribution. The value of a* in samples that have been treated with pressure and temperature increased significantly with increasing in the concentrations. Hayes and Kelly (2003) evaluated the effect of homogenizer pressure on properties of cow milk. They reported with an increase in homogenizer pressure from 0 to 200 bar; this value decreased significantly. The b* value in all samples decreased significantly with increasing in homogenizer pressure and temperature. Hayes and Kelly (2003) evaluated the effect of homogenizer pressure on properties of cow milk. They reported by increasing homogenizer pressure from 0 to 200 bar b* value decreased significantly. These observations of the color properties of treated samples in present study are in agreement with findings of research performed by Alvarenga et al. (2012), on production of vegetable cream using with green olive and soy which reported L*, a*, and b* of green olive; 66.75, −5.42, and 25.74, respectively.

**Table 2:** Effects of homogenizer pressure and temperature on color properties (L* (lightness), a* (redness–greenness), and b*(yellowness–blueness)) of cream

	Homogenizer pressure	Temperature	L*	b*	a*
1	125	45	61.67 ± 0.58bc	−4.00 ± 0.00b	33.67 ± 0.58a
2	125	60	61.00 ± 0.00 cd	−4.00 ± 0.00b	32.67 ± 0.58a
3	125	75	60.00 ± 1.00d	−2.00 ± 0.00a	30.67 ± 0.58b
4	145	45	63.33 ± 0.58a	−3.67 ± 0.58b	29.67 ± 0.58b
5	145	60	63.00 ± 0.00a	−3.3 ± 0.58b	29.67 ± 0.58b
6	145	75	63.00 ± 1.00a	−3.33 ± 1.15b	28.00 ± 1.73c
7	165	45	63.67 ± 0.58a	−1.33 ± 0.58a	26.33 ± 0.58d
8	165	60	63.00 ± 0.00a	−1.33 ± 0.58a	26.67 ± 0.58 cd
9	165	75	62.67 ± 0.58ab	−1.33 ± 0.58 a	26.67 ± 0.58 cd

Values within each column with different letters are significantly different (*p *<* *0.05).

The abbreviations are the same as in table.

All values are mean ± standard deviation.

### 3.4 Viscosity

The effects of homogenizer pressure and temperature on viscosity of cream are shown in Table 3. By evaluating the effect of temperature on *K*, the results showed treated sample in 60°C has the highest *K*. There was not any difference between 45 and 75°C treated samples. The results of pressure showed sample treated with 145 and 165 bar have the highest *K*, and there was no difference between this treatments. Long, Zhao, Zhao, Yang, and Liu (2012) evaluated the effects of homogenization on whipping cream. The results showed that by increasing pressure from 20 to 50 MPa, viscosity increased significantly. Also, with an increase in pressure, droplet size decreases significantly and number of droplet in water phase increases. This phenomenon reduced speed of droplet move. Moreover, reduction in droplet size increases the amount of protein in surface of cream droplet and increases viscosity.

**Table 3:** Effects of homogenizer pressure and temperature on droplet size and viscosity of cream

	Homogenizer pressure	Temperature	*n*	*K*	Droplet size (μm)	Span
1	125	45	0.39 ± 0.01b	393.70 ± 12.16d	0.551 ± 0.062	0.31
2	125	60	0.09 ± 0.00d	399.70 ± 1.27cd	0.549 ± 0.071	0.44
3	125	75	0.09 ± 0.01d	375.30 ± 0.42e	0.445 ± 0.079	0.47
4	145	45	0.27 ± 0.00c	420.40 ± 1.13b	0.446 ± 0.071	0.43
5	145	60	0.36 ± 0.02b	447.75 ± 10.96a	0.444 ± 0.076	0.46
6	145	75	0.27 ± 0.01c	414.30 ± 14.71bc	0.441 ± 0.070	0.50
7	165	45	0.36 ± 0.01b	421.85 ± 4.17b	0.437 ± 0.072	0.45
8	165	60	0.45 ± 0.01a	430.05 ± 1.91b	0.419 ± 0.063	0.39
9	165	75	0.36 ± 0.02b	428.70 ± 3.82b	0.409 ± 0.058	0.38

Values within each column with different letters are significantly different (*p *<* *0.05).

The abbreviations are the same as in table.

All values are mean ± standard deviation.

In evaluation of the effects of temperature on *n*, the result showed there was not a significant difference between samples treated with 45, 60, and 70°C but to increase pressure *n* decreased significantly. Innocente, Biasutti, Venir, Spaziani, and Marchesini (2009) evaluated the effect of homogenization on rheology properties of mixed ice cream. The results showed that by increasing pressure of homogenization viscosity increases significantly.

### 3.5 Droplet size distribution

The effects of different homogenizer pressure and temperature on the droplet size distribution and span of treatments are shown in Table 3. The prepared samples were diluted for 10 times and then analyzed to determine particle size distribution. Generally, mixed systems that consist of both emulsifier and stabilizer could more prolong the aggregation of particle compared to single‐type systems. By an increase in homogenizer pressure and temperature, amount of droplet size significantly decreased. Yuan, Gao, Zhao, and Mao (2008) produced nanoemulsion of β‐carotene and evaluated temperature and homogenizer pressure on properties of it. They reported by increasing temperature from 30 to 70°C droplet size decreased significantly; due to increase in velocity of oil phase and comfortable break of droplet, they also reported by an increase in pressure from 60 to 140 MPa droplet size decreased significantly. Increase in shear stress and intensity of fluid are the main reason of this effect. Long et al. (2012) evaluated the effect of homogenizer on whipping cream properties. They reported with an increase in pressure from 20 to 50 MPa droplet size decreased significantly, due to shear stress in high pressure.

### 3.6 Sensorial attributes

Sensory attributes are one of the most efficient tests to evaluate the quality change of products (Abedi, Naseri, Ghanbarian, & Vazirzadeh, 2016). Sensory attributes including texture, color, aroma, taste, and general acceptability of samples were evaluated, and results of their analysis are shown in Table 4. The aroma, taste, texture, and color in samples treated with 60°C were determined as appropriate samples by panelists. Results showed sample produced in 60°C and 145 bar pressure have the highest acceptability. Akhtar, Murray, and Dowu (2014) evaluated the effect of temperature on properties of mayonnaize sauce and cream sauce. The results showed that by increasing temperature taste and texture improved significantly.

**Table 4:** Effects of homogenizer pressure and temperature on sensorial attributes of cream

	Homogenizer pressure	Temperature	Texture	Aroma	Color	Taste	Acceptability
1	125	45	3.70 ± 0.82	3.70 ± 0.82	3.60 ± 0.52	3.60 ± 0.52	3.40 ± 0.70
2	125	60	3.60 ± 0.52	3.60 ± 0.79	3.90 ± 0.74	3.80 ± 0.79	3.60 ± 0.70
3	125	75	3.60 ± 0.70	3.60 ± 0.52	3.70 ± 0.48	3.70 ± 0.67	3.60 ± 0.52
4	145	45	4.10 ± 0.57	3.10 ± 0.63	4.00 ± 0.47	3.90 ± 0.57	3.90 ± 0.57
5	145	60	4.00 ± 0.94	4.00 ± 0.79	4.30 ± 0.48	4.20 ± 0.79	4.30 ± 0.82
6	145	75	3.90 ± 0.74	3.90 ± 0.74	3.90 ± 0.74	3.80 ± 0.79	4.00 ± 0.67
7	165	45	3.60 ± 0.70	3.60 ± 0.88	3.80 ± 0.70	3.80 ± 0.79	3.90 ± 0.88
8	165	60	4.00 ± 0.67	3.00 ± 0.74	4.10 ± 0.57	4.10 ± 0.74	3.80 ± 0.79
9	165	75	3.60 ± 0.52	3.60 ± 0.63	3.70 ± 0.48	3.60 ± 0.52	3.60 ± 0.52

Values within each column with different letters are significantly different (*p *<* *0.05).

The abbreviations are the same as in table.

All values are mean ± standard deviation.

## 4 CONCLUSION

This study helped to increase the knowledge about properties changes in vegetable cream under different conditions of processing. Different vegetable creams were homogenized at different pressures and temperatures. Rheological properties indicated that increasing the pressure brought about the cream from a shear‐thinning behavior to a power low behavior, with a great increase in the viscosity. Moreover, the amount of pressure markedly effects on droplet size distributions. The high shear, high pressure, and relatively high temperature could also damage some constituents and properties of vegetable cream, that is why the experimental evaluation needs to be extended to give a more realistic and precise description of cream formation in the high‐pressure homogenization method. In addition, systematic test application well‐characterized emulsions and homogenization method need to be carried out.

## ETHICAL STATEMENT

In this research, we used humans only for test panelist and I ensure this research conducted following the principles of the Institute of Standards and Industrial Research of Iran.

## CONFLICT OF INTEREST

None declared.
